# Adsorptive Treatment of Cr (VI)-Contaminated Wastewater in a Fixed-Bed Column Using Hydrothermal Chitosan/Polyvinyl Alcohol Beads and Life Cycle Assessment

**DOI:** 10.3390/polym18101167

**Published:** 2026-05-09

**Authors:** Eylul Kosoglu, Asude Sena Demirci Ulke, Yasar Andelib Aydin

**Affiliations:** Chemical Engineering Department, Faculty of Engineering, Marmara University, Maltepe, Istanbul 34854, Türkiye; eylulkosoglu@marun.edu.tr (E.K.); asude.ulke@marmara.edu.tr (A.S.D.U.)

**Keywords:** hydrothermally treated CS/PVA beads, fixed-bed column, continuous Cr (VI) adsorption, breakthrough curve, non-linear regression

## Abstract

Hydrothermally treated chitosan/polyvinyl alcohol beads (H-CS/PVA) were used as filler material in a fixed-bed column for continuous Cr (VI) removal. The effects of main operational parameters, namely bed height, initial concentration and flow rate, were evaluated in the respective ranges of 2–6 cm, 20–60 mg/L and 2.5–7.5 mL/min. Maximum removal efficiency and adsorption capacity were calculated as 64.2% and 15.53 mg/g, respectively. The corresponding breakthrough curves were analyzed by Yoon–Nelson, Adams–Bohart, Thomas and BDST (Bed Depth–Service Time) models, out of which the highest consistency was achieved with the Yoon–Nelson model for all studied conditions. The adsorbent maintained strong reusability, showing minimal loss (~2.5%) in desorption efficiency across three successive regeneration cycles with 0.1 M NaOH as the eluent. SEM and SEM–EDX analyses confirmed the presence of chromium on the H-CS/PVA surface at an elemental fraction of 1.03% (w.). Furthermore, FTIR and XPS analyses verified the role of amine and hydroxyl functionalities in the complexation and adsorption of Cr (VI). Overall, a column system operated under optimal conditions (H_bed_: 6 cm, C_0_: 40 mg/L, and column diameter: 2.5 cm) and regenerated three times can efficiently treat 20 L of Cr (VI)-contaminated wastewater, resulting in an associated environmental impact of 0.896 kg CO_2_-eq.

## 1. Introduction

Classified as a Group A carcinogen by the International Agency for Research on Cancer (IARC), Cr (VI) is one of the most dangerous heavy metal pollutants due to its high solubility and mobility in water and high toxicity [1]. Cr (VI) released into aquatic environments as a result of industrial activities such as electroplating, leather tanning, textile dyeing, and pigment production causes serious adverse effects on human health and ecosystems even at low concentrations [2]. Therefore, the effective removal of Cr (VI) from wastewater is of great importance.

Among existing treatment methods, adsorption is widely preferred due to its high removal efficiency, simple operation, and cost-effectiveness. In recent years, biopolymer-based adsorbents have attracted attention due to their biodegradable and environmentally friendly properties [3]. Chitosan (CS), a derivative of chitin, exhibits high affinity for Cu^2+^, Pb^2+^, Cd^2+^, Zn^2+^, Ni^2+^, Hg^2+^, and Cr (VI) metal ions due to the -NH_2_ (amino) and -OH (hydroxyl) groups in its structure, making it a promising adsorbent [4,5,6]. However, the weak mechanical strength and instability of pure chitosan in acidic environments limit its use [7]. It is common practice to use crosslinking agents and blends with other polymers or form composite structures of CS that enhance mechanical strength, chemical stability, and resistance to acidic environments, thereby improving suitability for practical and continuous adsorption applications. Among various polymers such as alginate [8], polyethyleneimine (PEI) [9], polyethylene glycol (PEG) [10], polyvinylpyrrolidone (PVP) [11], cellulose derivatives [8], and polylactic acid (PLA) [12] that have been explored to improve the mechanical stability of CS-based adsorbents, polyvinyl alcohol (PVA) is particularly favored due to its low cost, large-scale industrial availability, excellent compatibility with CS, non-toxic nature, and comparatively low environmental impact, making it highly suitable for use in aqueous systems [13,14]. Various forms of such composites have been prepared, including films, hydrogels, foams, membranes, etc. [15,16]. However, specifically tailored adsorbents in spherical morphology provide significant advantages in terms of mass transfer related to microporous structure in addition to ease of recovery and regeneration in a column system [17,18,19]. Studies reported in the literature to reveal the properties of CS-based polymeric adsorbents for various heavy metals developed with different counterparts have been compiled and are presented comparatively in Table 1.

The structural integrity and stability of the composite are assured by inter and intra-molecular hydrogen bonds as well as chemical crosslinks formed between CS and PVA molecules by either physical and/or chemical crosslinking [20,21]. Though chemical crosslinking assures satisfactory mechanical and physical endurance, it also leads to a loss of active adsorption sites. Moreover, common chemical crosslinking agents such as glutaraldehyde, epichlorohydrin and ethylene glycol diglycidyl ether have well-established toxic effects. Thus, the development of effective physical cross-linking procedures is required to eliminate the requirement for chemical crosslinking. Yi et al. (2020) have proposed hydrothermal treatment as a beneficial method for increasing the acid stability of CS without losing its functional groups, hence contributing to the enhancement of Cr (VI) adsorption capacity under batch conditions [22].

**Table 1:** The literature overview of CS-based blend or composite adsorbents with different morphologies.

Sample Name	Morphological Structure	Metal Ion	Adsorption Capacity (mg/g)	Experimental System	Ref.
CS/PVA	Film	Zn (II)	7.816	Batch	[23]
CS/PVA	Membrane	Cu(II)	112.13	Batch	[23]
CS/PVA/PEI	Membrane	Ni (II)	86.08	Batch	[24]
CS/PVA/PEI	Membrane	Cd (II)	75.5	Batch	[24]
CS/PVA	Film	Pb (II)	7.67	Batch	[25]
CS/PVA/a-MWCNT	Film	Cr (VI)	134.2	Batch	[26]
CS/PVA	Film	Cr (VI)	49.34	Batch	[26]
CS coated kaolin	Bead	Cr (VI)	31.98	Batch	[27]
CS/SDS ^1^	Bead	Cr (VI)	3.23	Batch	[28]
CS/MWCNT ^2^/Fe	Bead	Cr (VI)	1.54	Fixed-bed	[29]
PE/Agave/CS	Pellet	Cr (VI)	9.7	Fixed-bed	[30]
Silanized CS/PE/PS ^3^/Agave	Pellet	Cr (VI)	12	Fixed-bed	[31]
CS/Coal Fly Ash	Nanopowder	Cr (VI)	42.08	Fixed-bed	[32]
CS/CRH ^4^	Bead	Cr (VI)	52.5	Fixed-bed	[33]
CS/PVA	Bead	Cr (VI)	33.09	Batch	[34]
H ^5^-CS/PVA	Bead	Cr (VI)	33.09	Batch	[34]

^1^ Sodium dodecyl sulphate; ^2^ multi-walled carbon nanotube; ^3^ polystyrene; ^4^ carbonized rice husk; ^5^ hydrothermally treated.

In our previous work, a hydrothermal treatment technique was employed in the synthesis of CS/PVA beads, and the beads were utilized in batch adsorption of Cr (VI). As referred to in Table 1, the adsorption capacity of H-CS/PVA beads outperformed chemically cross-linked beads in terms of Cr (VI) adsorption capacity due to multilayer adsorption controlled by an intra-particle diffusion mechanism. Notable enhancements were also detected in the surface area, pore characteristics, chemical structure and mechanical durability when the treatment was conducted under optimal operation conditions of 140 °C and 12 h [34]. The results of these works clearly verified the effectiveness of H-CS/PVA beads in Cr (VI) adsorption under batch conditions and highlighted the need to evaluate their performance in continuous fixed-bed column systems, where the dynamics and breakthrough characteristics can be investigated.

Despite the fact that fixed-bed column systems better represent actual wastewater treatment processes and enable the determination of critical parameters for scale-up, heavy metal adsorption has mostly been evaluated in batch systems, and remediation performance under continuous flow conditions has been relatively less studied in the literature [35]. In fixed-bed column systems, key design and operational parameters such as bed height, flow rate, influent concentration, contact time, and mass transfer zone characteristics play a critical role in the determination of adsorption efficiency and are essential for accurate scale-up and process optimization [19,36].

Although CS-based adsorbents and fixed-bed Cr (VI) removal systems are widely reported, hydrothermally stabilized, cross-linker-free materials in column applications still remain limited. In this context, this study focused on the utilization of H-CS/PVA composite beads as a packing material in a fixed-bed column for the removal of Cr (VI) from aqueous solutions. The effects of operating parameters, i.e., flow rate, initial concentration, and bed height, on breakthrough behavior were systematically investigated. Furthermore, the experimental data were evaluated using column models of Thomas, Yoon–Nelson, and BDST to characterize the fracture behavior of the fixed-bed adsorption column, determine the kinetic parameters, and reveal the scale-up potential of H-CS/PVA beads for continuous Cr (VI) removal. Moreover, a hydrothermal synthesis approach was combined with LCA to evaluate both adsorption performance and environmental impact. Therefore, the present study bridges the gap between laboratory-scale optimization and continuous process performance, offering a unique contribution to full-scale wastewater treatment applications.

## 2. Materials and Methods

### 2.1. Materials

Chitosan (MW 100,000–300,000 g/mol, with 85% deacetylation degree) and polyvinyl alcohol (98–99%) were purchased from Sigma-Aldrich (St. Louis, MO, USA) and Acros (Geel, Belgium), respectively. Potassium chromate was obtained from Merck (extra pure, Darmstadt, Germany). 1,5-diphenyl carbazide (extra pure, Sigma-Aldrich) and acetone (≥99.0%, Carlo Erba, Heudebouville, France) were used for the indicator solution. Sodium hydroxide (>98%), hydrochloric acid (36.5–38%) and sulfuric acid (96–97%) were purchased from Sigma-Aldrich (Schnelldorf, Germany).

### 2.2. Methods

#### 2.2.1. Synthesis of H-CS/PVA Adsorbents

**Figure 1:**
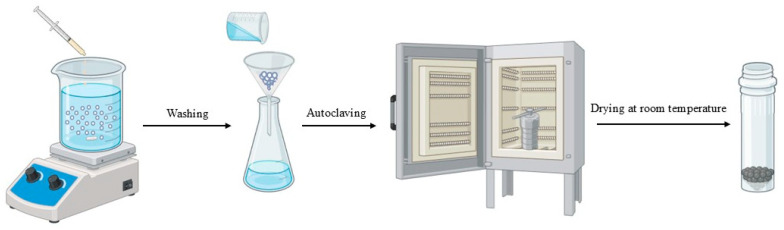
H-CS/PVA adsorbent beads synthesis procedure.

CS (4% *w*/*v*, in acetic acid solution) and PVA (10% *w*/*v*, in pure water) solutions were prepared and mixed in equal volumetric ratio. A pump-syringe system (Lead Fluid/Nanbei, BT600F, Baoding, China) was used to transfer this mixture into a 1 M NaOH bath. Afterwards, the hydrogels were filtered and washed rigorously with pure water for the removal of excess NaOH. The hydrogels were taken into a stainless-steel autoclave and subjected to thermal treatment at 140 °C for 12 h. As the last stage of the synthesis, the carbonized beads were left to dry at room temperature. The synthesis procedure of H-CS/PVA adsorbent beads is depicted in Figure 1.

#### 2.2.2. Installation of the Fixed-Bed Adsorption Column

Cr (VI) removal was fulfilled in a fixed-bed column. The experimental configuration comprised a column including a fixed bed filled with H-CS/PVA beads, a peristaltic pump, a feed solution tank, and a receiver tank for the effluent stream. A 2.5 cm inner diameter cylindrical glass column with a Teflon tap was used to install the continuous flow column.

### 2.3. The Effect of Operational Parameters Investigation on Continuous Cr (VI) Removal

The effects of wastewater flow rate, initial Cr (VI) concentration and bed height on the adsorption behavior were systematically investigated in the corresponding ranges of 2.5–7.5 mL/min, 20–60 mg/L, and 2–6 cm, respectively. The pH of the influent stream was maintained at pH 3.0 in all experiments based on preliminary batch results indicating a marked decrease in adsorption capacity at pH > 3.0 [34]. The decrease is mainly related to (i) partial deprotonation of amine functional groups leading to reduced electrostatic attraction toward HCrO_4_^−^ ions and (ii) the takeover of CrO_4_^2−^ ions instead of HCrO_4_^−^, which requires an additional adsorption site due to its charge [34,37]. The Cr (VI) concentration of the effluent was monitored during the experiments by taking samples at predetermined time intervals. Experiments were performed until the effluent reached the initial concentration. The concentration of Cr (VI) was determined via colorimetric analysis using a 1–5,diphenyl carbazide reagent as the complexing agent spectrophotometrically at 540 nm (Shimadzu UV 1240, Shimadzu, Kyoto, Japan) [26,34].

For each case, breakthrough curves were constructed and analyzed to evaluate the column performance under different operating conditions and to determine the optimal parameters for efficient Cr (VI) removal. During flow rate experiments, C_0_ (20 mg/L) and h_bed_ (2 cm) were kept constant; during concentration studies, Q (2.5 mL/min) and h_bed_ (2 cm) were fixed; and during bed height tests, Q (5 mL/min) and C_0_ (40 mg/L) were maintained to evaluate column performance and breakthrough behavior.

### 2.4. Mathematical Modeling Studies on Fixed-Bed Column

Thomas, Adams–Bohart, Yoon–Nelson and BDST models (Equations (1)–(4)) were employed to explain the adsorption mechanism and determine the appropriate model. Non-linear regression analysis was performed using the Microsoft 365 Excel Solver tool (WA, USA) in model fitting to adsorption data.

(1) $$\ln(\frac{C_{t}}{C_{0}}-1)=\frac{k_{TH}q_{0}m}{Q}-\frac{k_{TH}C_{0}V_{eff}}{Q}$$

(2) $$\ln(\frac{C_{t}}{C_{0}})=k_{AB}C_{0}t-k_{AB}N_{0}\frac{z}{U_{0}}$$

(3) $$\ln(\frac{C_{t}}{C_{0}-C_{t}})=k_{YN}t-k_{YN}\tau$$

(4) $$t=\frac{N_{0}Z}{C_{0}u}-\frac{1}{k_{BDST}C_{0}}\;\ln(\frac{C_{0}}{C_{t}}-1)$$

In the Thomas, Adams–Bohart, Yoon–Nelson, and BDST models, $C_{0}$ represents the influent (initial) adsorbate concentration (mg/L), while $C_{t}$ denotes the effluent concentration at time t (mg/L), and t is the contact time. In the Thomas model, $k_{TH}$ is the Thomas rate constant (mL·mg^−1^·min^−1^), $q_{0}\;$is the maximum adsorption capacity of the adsorbent (mg/g), m is the mass of adsorbent packed in the column (g), Q is the volumetric flow rate, and $V_{eff}$ is the effective volume of the treated solution. In the Adams–Bohart model, $k_{AB}$ denotes the kinetic constant, $N_{0}\;$is the maximum volumetric adsorption capacity of the adsorbent (mg/L), z represents the bed height (cm), and $v_{0}$ is the superficial velocity (cm/min). For the Yoon–Nelson model, $k_{YN}$ is the rate constant (min^−1^) and τ is the time required for the effluent concentration to reach 50% of the influent concentration. In the BDST model, $N_{0}$ indicates the adsorption capacity per unit bed volume, z is the bed depth, v is the linear flow velocity, and $k_{BDST}$ is the BDST rate constant [38,39,40,41].

To identify the most appropriate kinetic model, a comprehensive error analysis was performed. The discrepancies between the experimentally obtained data and the values predicted by the applied models were quantified, and the magnitude of error was calculated for each individual data point. For this purpose, the non-linear chi-square test (*x*^2^), Average Relative Error (*ARE*), and Marquardt’s Percent Standard Deviation (*MPSD*) were employed as statistical criteria to evaluate model performance. The equations for the non-linear chi-square test, Average Relative Error and Marquardt’s Percent Standard Deviation models are given in Equations (5)–(7), respectively.

(5) $$X^{2}=\sum_{İ=1}^{N}\frac{{\left(q_{e,measurement}-q_{e,calculated}\right)}^{2}}{q_{e,measurement}}$$

(6) $$\mathrm{ARE}=\frac{100}{n}\sum_{İ=1}^{N}\frac{\;q_{e,measurement\;}-q_{e,calculated}}{q_{e,measurement\;}}$$

(7) $$\mathrm{MPDS}=100\sqrt{\frac{1}{n-p}\sum_{İ=1}^{N}\frac{{\left(q_{e,measurement}-q_{e,calculated}\right)}^{2}}{q_{e,measurement}}}$$

### 2.5. Characterization

SEM–EDX analyses were conducted at Namık Kemal University Research Lab (NABILTEM) with a FEI Quanta Feg 250 instrument (Thermo Fisher Scientific, Waltham, MA, USA) and a Bruker Vertex 70 ATR instrument (Bruker Corporation, Ettlingen, Germany), respectively. The structural analysis of hydrogel beads was performed by Fourier transform infrared spectroscopy (FT-IR) at Marmara University Research Laboratory using a TG/DTG-Q50 apparatus (TA Instruments, New Castle, DE, USA) within the wavenumber range of 4000–500 cm^−1^. The surface area and pore volume of adsorbent beads were measured by the technique of the physical adsorption of nitrogen gas at 77 K. The analysis was performed using a Quantachrome NovaWin2 device (Quantachrome Instruments, Boynton Beach, FL, USA) in Yildiz Technical University, MERLAB. Samples were degassed at 378 K for 8 h prior to analysis. XPS analysis of the exhausted H-CS/PVA beads after Cr (VI) adsorption was carried out at the Advanced Research Laboratory of Bogazici University using a Thermo Scientific K-Alpha XPS System (Thermo Fisher Scientific) equipped with a monochromatic Al Kα X-ray source to elucidate the Cr (VI) adsorption mechanism.

### 2.6. Regeneration Studies

Bead regeneration and reuse studies were conducted using the same setup as the fixed-bed column system employed in the adsorption experiments. Prior to column regeneration experiments, preliminary batch desorption tests were performed using different eluents, including EDTA (ethylenediaminetetraacetic acid), HCl, and NaOH, in order to identify the most effective desorption agent. The identified desorption solution was passed through the column, and the desorbed Cr (VI) concentration was assessed at predetermined time intervals. Following desorption, the column was washed with deionized water for pH restoration. These adsorption–desorption cycles were repeated over a total of 3 times to evaluate the adsorbent’s regeneration performance and reusability.

### 2.7. Life Cycle Analysis

The Life Cycle Assessment (LCA) approach was used to evaluate the environmental impacts of producing H-CS/PVA adsorbent beads required for the treatment of Cr (VI)-contaminated water. The assessment covered raw material acquisition, chemical use, production procedures, and electricity consumption required for the operations run under optimized conditions. The system boundary was defined as “cradle-to-use.” The Ecoinvent (v3.11, Zurich, Switzerland) and SimaPro 9.0 programs (LE Amersfoort, The Netherlands) provided the dataset. Where possible, the Ecoinvent was utilized to model the chemicals employed in the process, while for some chemicals, alternate literature sources were used.

## 3. Results and Discussion

### 3.1. Investigation of the Effect Parameters in Continuous Cr (VI) Removal in a Fixed-Bed Column

**Figure 2:**
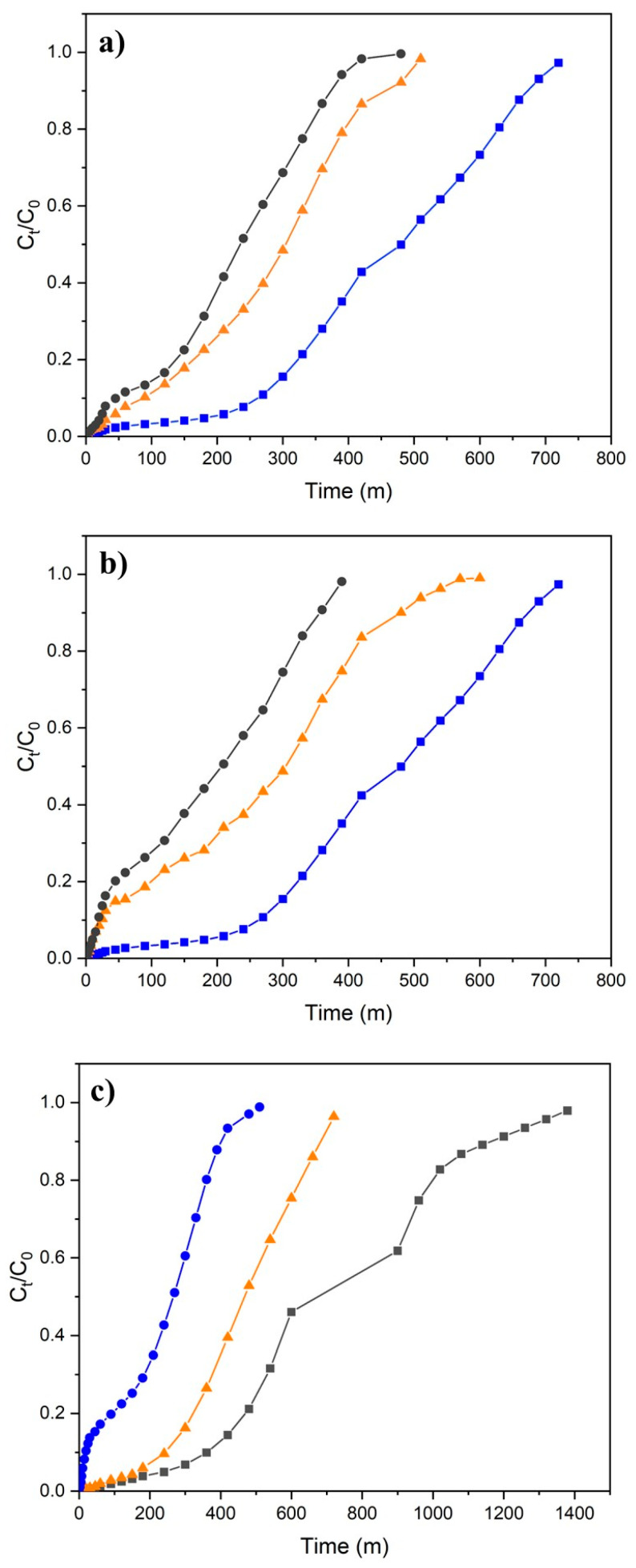
Effect of process parameters on breakthrough behavior (**a**) at different flow rates (blue (■), orange (▲), and gray (●) represent 2.5, 5, and 7.5 mL/min); (**b**) at different initial concentrations (blue (■), orange (▲), and gray (●) represent 20, 40, and 60 mg/L); and (**c**) at different bed heights (blue (●), orange (▲) and gray (■) represent 2, 4, and 6 cm, respectively).

Breakthrough curves define dynamic adsorption behavior by revealing the relationship between outlet concentration and operating time in fixed-bed columns; thus providing fundamental information for column design, service life estimation, and scale-up studies of continuous adsorption systems. The performance in a fixed-bed column is strongly influenced by operating and design parameters [42,43]. The effects of key operating parameters, including flow rate (Q), bed height (h_bed_), and inlet concentration (C_0_), on the performance of the fixed-bed column were systematically investigated. Breakthrough curves (BCs) were obtained by varying one parameter at a time while keeping the others constant. The resulting BCs and corresponding column parameters are presented in Figure 2 and Table 2.

The breakthrough curves in Figure 2a indicate that increasing the flow rate leads to earlier breakthrough and faster saturation of the column due to reduced contact time between Cr (VI) and H-CS/PVA. Although higher flow rates allow for the treatment of a larger volume of solution, the adsorption and bed utilization efficiency have been reported to decrease significantly, attributed mainly to the reduction in residence time, which limits the time for binding of the Cr (VI) molecules to the H-CS/PVA surface [35,44,45].

The effect of initial concentration is visualized in Figure 2b. Accordingly, the saturation time decreases significantly at high concentrations due to the fact that the increased driving force causes the active sites on the adsorbent to fill more rapidly and the mass transfer zone to narrow, resulting in the breakthrough curve shifting to the left with a steeper slope. Concordantly, the column can operate for a much longer period (approximately +700 min) at low concentrations, and as the concentration increases, the operating time is reduced by half, approximately. The sharpening of the breakthrough curves might be interpreted as an indication of a narrowed mass transfer zone [35].

Upon examining Table 2, it is observed that the increase in initial concentration has two fundamental conflicting effects on column performance: a reduction in operational lifetime and an increase in unit capacity. When the concentration increases from 20 to 60 mg/L, the active regions rapidly fill up due to the increased contaminant load, and the column’s exhaust time drops from 250 min to 20 min, operationally limiting the system’s efficiency substantially. On the other hand, the strong mass transfer driving force created by high concentration provides more effective diffusion into the pores of the H-CS/PVA adsorbent, increasing the amount of material retained per gram by more than 50%. Consequently, although the chemical potential of the adsorbent is utilized more effectively in the treatment of high-concentration wastewater, more frequent adsorbent replacement or a higher bed height is required to ensure the continuity of the system [41,43].

When considering the effect of bed height as well, increasing the bed height (Figure 2c) significantly delays the breakthrough time by increasing the total amount of adsorbent in the column and, consequently, the number of available active sites. At low bed heights, the contaminant molecules have a short contact time with the adsorbent, causing the column to reach saturation rapidly [46]. As the bed height increases, the residence time of the wastewater in the column increases and the mass transfer zone spreads over a wider area, extending the operational life of the system to over 1400 min. This proves that increasing the bed height is the most critical parameter for maximizing the volume of water treated per unit time and the service life of the column.

### 3.2. Model Studies on Fixed-Bed Adsorption Column

**Figure 3:**
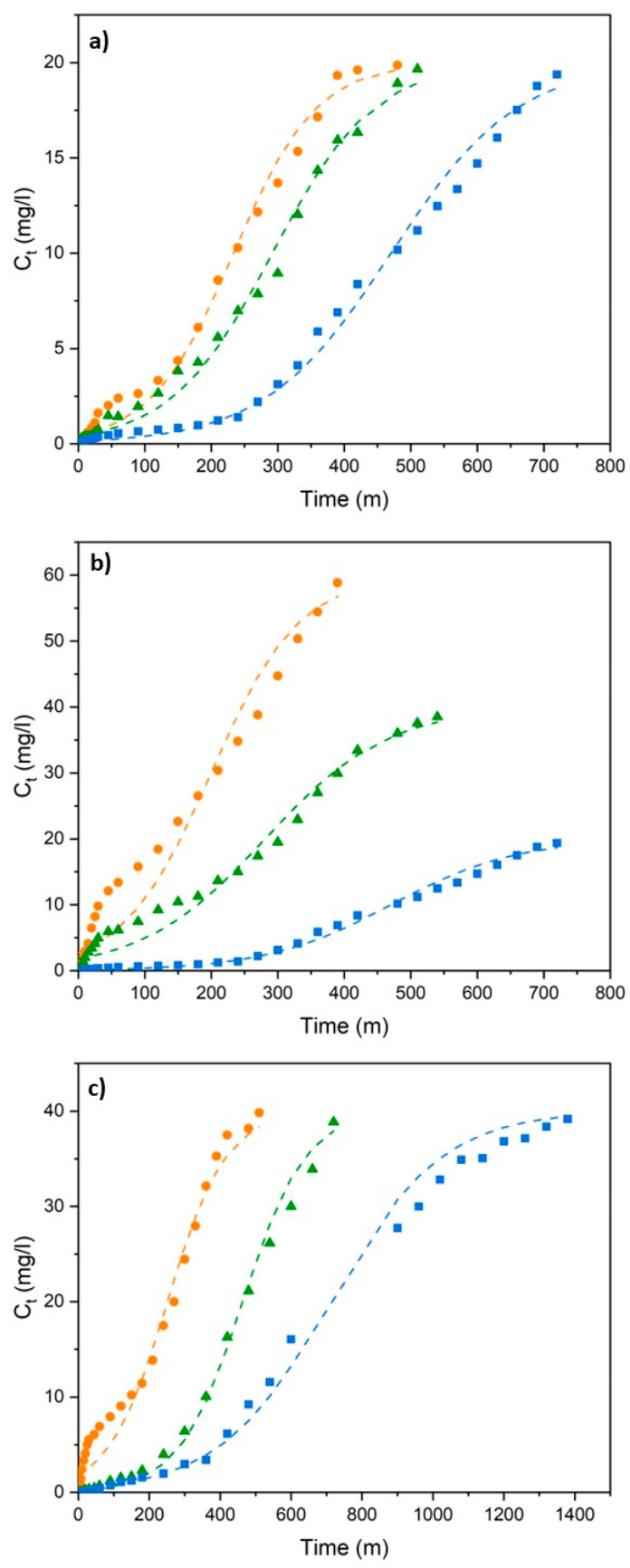
Thomas model fitting of breakthrough curves for (**a**) different flow rates (blue (■), green (▲), and orange (●) represent 2.5, 5 and 7.5 mL/min, respectively, at h_bed_ = 2 cm and C_0_ = 20 mg/L); (**b**) different initial concentrations (blue (■), green (▲), and orange (●) represent 20, 40, and 60 mg/L, respectively, at h_bed_ = 2 cm and Q = 2.5 mL/min); and (**c**) different bed heights (blue (■), green (▲), and orange (●) represent h_bed_ = 6, 4, and 2 cm, respectively, at Q = 5 mL/min and C_0_ = 40 mg/L).

**Figure 4:**
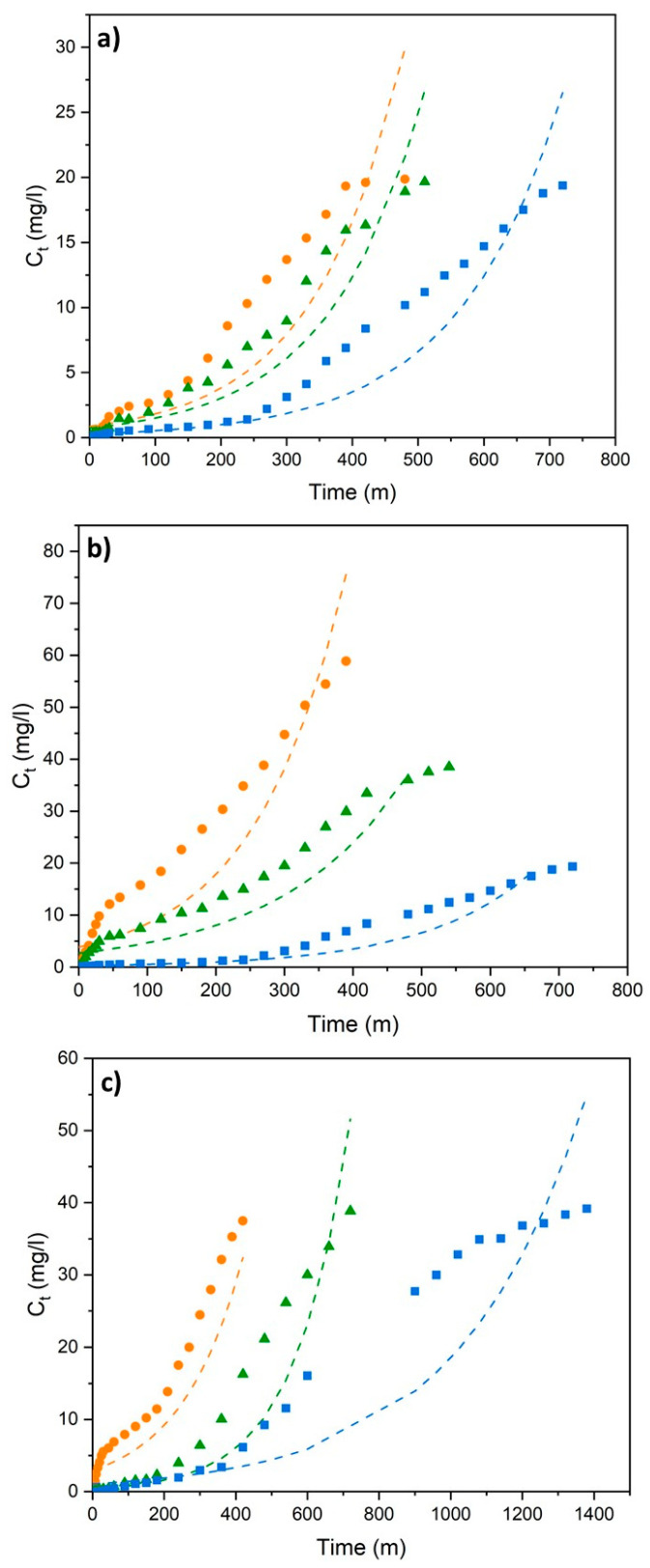
Adams–Bohart model fitting of breakthrough curves for (**a**) different flow rates (blue (■), green (▲), and orange (●) represent 2.5, 5, and 7.5 mL/min, respectively, at h_bed_ = 2 cm and C_0_ = 20 mg/L); (**b**) different initial concentrations (blue (■), green (▲), and orange (●) represent 20, 40, and 60 mg/L, respectively, at h_bed_ = 2 cm and Q = 2.5 mL/min); and (**c**) different bed heights, (blue (■), green (▲), and orange (●) represent 6, 4, and 2 cm, respectively, at Q = 5 mL/min and C_0_ = 40 mg/L).

**Figure 5:**
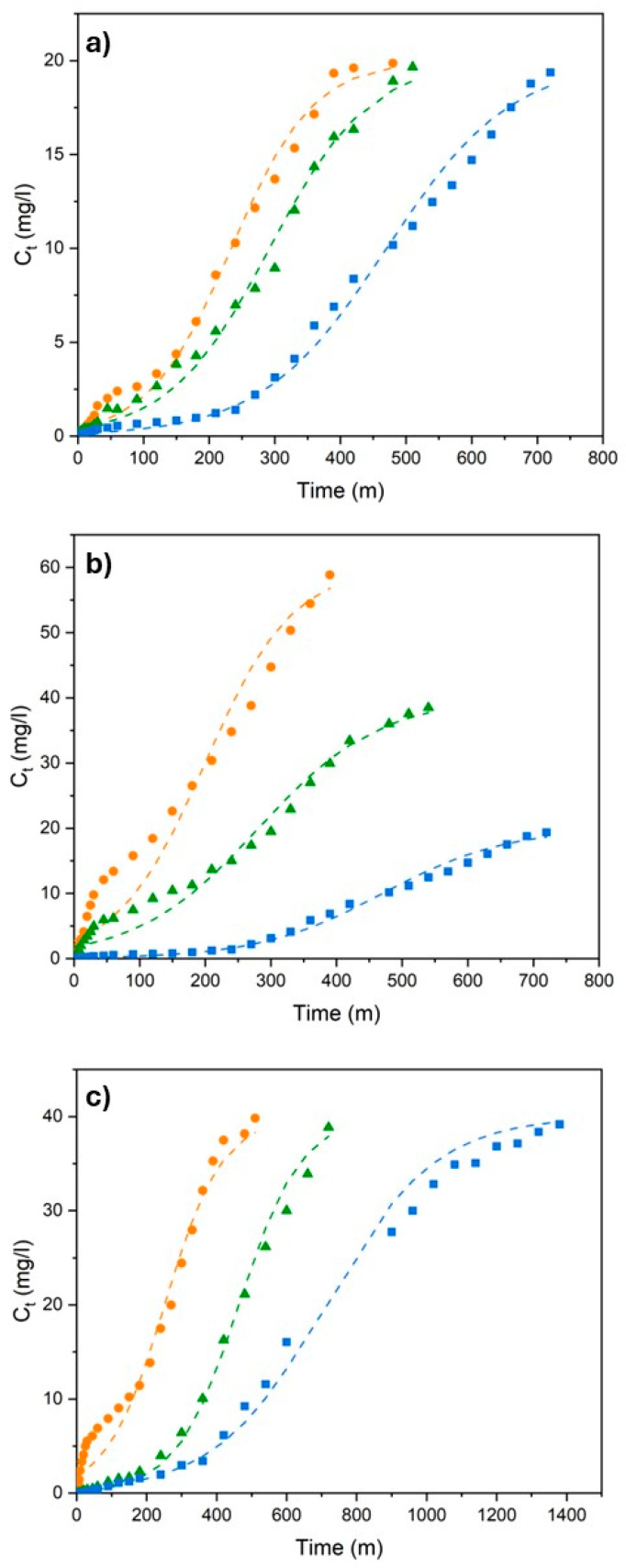
Yoon–Nelson model fitting of breakthrough curves for (**a**) different flow rates (blue (■), green (▲), and orange (●) represent 2.5, 5, and 7.5 mL/min, respectively, at h_bed_ = 2 cm and C_0_ = 20 mg/L); (**b**) different initial concentrations (blue (■), green (▲), and orange (●) represent 20, 40, and 60 mg/L, respectively, at h_bed_ = 2 cm and Q = 2.5 mL/min); and (**c**) different bed heights (blue (■), green (▲), and orange (●) represent 6, 4, and 2 cm, respectively, at Q = 5 mL/min and C_0_ = 40 mg/L).

The Thomas, Adams–Bohart, and Yoon–Nelson models were applied to analyze the breakthrough curves, evaluate the column performance and discuss specific aspects of the adsorption process, such as adsorption kinetics, bed capacity, and breakthrough time. The results obtained at different flow rates, initial concentrations, and bed heights are shown in Figure 3, Figure 4 and Figure 5 and Table 3.

When the results of the Thomas model kinetic analysis are evaluated overall, it is observed that the increased flow rate reduces the mass transfer resistance, thereby increasing the rate constant (k_Th_), but shortens the contact time. Although an increase in the initial concentration triggers the driving force in the system, thereby increasing the theoretical adsorption capacity (q_0_), it also causes the adsorption rate to slow down relatively due to the rapid filling of the active sites. This behavior is generally expected in adsorption systems and has been observed in numerous studies, including those by Chen et al. [47], Dawood and Sen [48], Al-Othman et al. [49] and Sun et al. [50]. In most relevant examples, it has been reported that higher initial concentrations increase the theoretical adsorption capacity but prolong the time to reach equilibrium. Increasing the bed height maximizes the total amount of beads in the system and, consequently, the total adsorption capacity, achieving the highest theoretical capacity value (14.17 mg/g) at a bed height of 6 cm. The high deviation observed for the Thomas model, in some cases exceeding 100%, indicates limited applicability under the present experimental conditions. This is likely due to the ideal assumptions of the model not fully representing real column behavior, and therefore, the results should be interpreted together with other kinetic models for a more reliable evaluation.

The Adams–Bohart model is a mathematical model used to explain the kinetic behavior of a fixed-bed column, particularly during its initial phase of operation (the start-up region). Even though model applicability is limited to the early phase of the breakthrough curve, it was employed to evaluate the initial adsorption dynamics and provide insight into the onset of bed saturation. Its primary purpose is to calculate the system’s maximum adsorption capacity and rate constant, thereby predicting how long it will take for the column to reach its breakpoint under different operating conditions. This model enables the analysis of the saturation rate of the adsorbent bed, thereby providing the fundamental parameters required for industrial-scale column designs [51]. The Adams–Bohart model analysis results demonstrate that the kinetic behavior of the system in its initial phase has been successfully characterized under specific operating conditions, particularly with a low error margin (2.22%). Upon examination of the data, it is understood that an increase in flow velocity accelerates mass transfer (k_AB_), thereby increasing the rate constant; however, as the initial concentration and bed height increase, this coefficient decreases, causing the reaction to spread. While the volumetric capacity (N_0_) of the system increases in parallel with the increasing contaminant load and bed depth, the fact that the theoretical breakthrough times calculated by the model are higher than the experimental values proves that the Adams–Bohart model provides an optimistic but generally descriptive framework for the initial stages of the column.

Another kinetic approach used to describe adsorption behavior in fixed-bed column systems in a simple and practical manner is the Yoon–Nelson model. Its fundamental assumption is that the probability of the adsorbate passing through the bed is directly proportional to the probability of the adsorbate being retained [52]. The model explains the system’s performance based on only two parameters: the Yoon–Nelson rate constant (k_YN_) and the 50% breakthrough time (i.e., the time when C/C_0_ = 0.5) (τ). As shown in Table 3, the k_YN_ values increased with increasing flow rate (Q), while the 50% breakthrough time decreased. This indicates that adsorption occurs more rapidly at high flow rates due to the solution spending less time in the fixed bed, but the column reaches saturation more quickly. A similar trend was also observed when the initial concentration was increased; higher inlet concentrations accelerated the adsorption kinetics by creating a greater driving force but shortened the breakthrough time. Conversely, when the bed height was increased, the opposite behavior occurred: k_YN_ values decreased while the 50% breakthrough time increased. This result indicates that in deeper beds, the contaminant is retained in the column for a longer time due to the increased adsorbent amount and contact time. The low error percentages (ε < 6%) between the τ_cal_ values obtained from the model and the experimental τ_exp_ values demonstrate that the Yoon–Nelson model can predict system behavior with sufficient accuracy and successfully represent column performance.

Increasing the bed height maximizes the service life and capacity (maximum 15.53 mg/g) of the column, while increasing the flow rate and initial concentration rapidly shortens the saturation time. Kinetic analyses conducted using experimental data revealed that the Yoon–Nelson model best explains the system’s behavior with the lowest margin of error and highest accuracy, thus effectively predicting the breakthrough curves for different operation parameters. In particular, high bed height and low flow rates increased column performance and led to significant changes in the Yoon–Nelson parameters, agreeing with findings reported by Lissy et al., who studied the modeling of Cr (VI) adsorption in a fixed-bed of pyrrole-coated nanocomposites and differentiated this model due to its substantiated accuracy of fit with respect to Adams–Bohart and Thomas models [53].

Compliance with the Yoon–Nelson model suggested that the adsorption rate is proportional to the remaining adsorbent capacity. The breakpoint curve progresses symmetrically when half of the column capacity is reached and the breakpoint time is predictable [54].

#### BDST Model

The Bed Depth–Service Time (BDST) model was applied to evaluate the performance of a fixed-bed column for Cr (VI) removal using H-CS/PVA and to determine the dynamic adsorption parameters. The calculated BDST model parameters obtained under different operating conditions are presented in Table 4.

**Figure 6:**
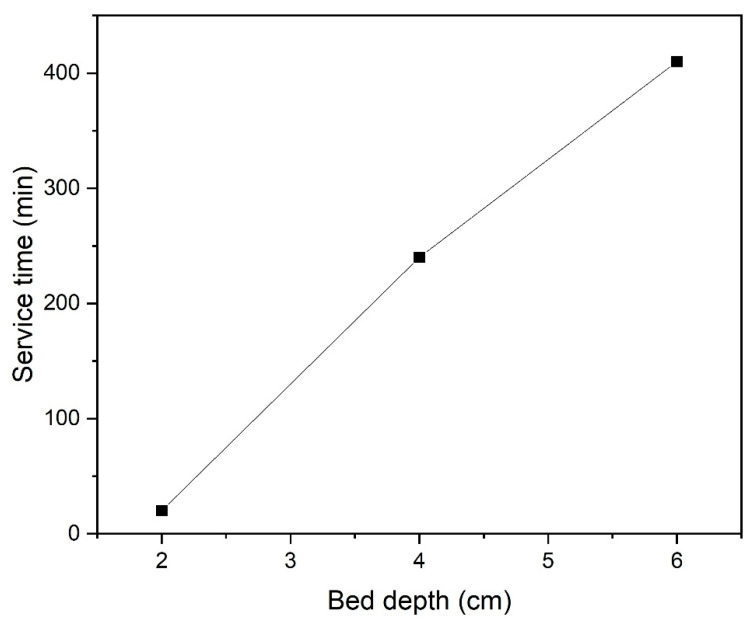
BDST model fit for Cr (VI) adsorption at C_0_ = 40 mg/L, Q = 5 mL/min and C_t_/C_0_ = 0.1.

Figure 6 shows the breakthrough time corresponding to the breakthrough criterion C_t_/C_0_ = 0.1 as a function of bed depth (h_bed_) under conditions of initial Cr (VI) concentration 40 mg/L and flow rate 5 mL/min.

A linear relationship with a high correlation coefficient (R^2^ = 0.995) was obtained between the service time and the bed depth, indicating that the BDST model adequately describes the Cr (VI) adsorption behavior in a fixed-bed column. The parameters of the BDST model are summarized in Table 5.

As seen in Table 5, the kinetic constant (k_BDST_) and adsorption capacity (N_0_) were calculated as 0.013 mL/mg·min and 24,075 mg/L, respectively, from the slope and intercept values of the BDST line. Furthermore, the minimum bed depth required to prevent sudden breakage was determined to be 1.7 cm based on the calculations. The high N_0_ value obtained demonstrates the strong affinity of H-CS/PVA towards Cr (VI), which can be attributed to the porous structure of the composite foam and the rich functional groups it contains. These parameters provide important information for preliminary column design and scale-up studies. Although the BDST model offers a practical approach for estimating column performance, deviations between experimental and calculated service times have been observed under different operating conditions. This indicates that the model is more suitable for initial design calculations rather than making accurate predictions over a wide range of flow rates and inlet concentrations. Overall, the BDST analysis confirms that H-CS/PVA is an effective adsorbent for continuous Cr (VI) removal and highlights its potential in fixed-bed applications.

### 3.3. Error Analysis

Dynamic modeling of the fixed-bed adsorption column was performed using the Thomas, Adams–Bohart, Yoon–Nelson, and BDST models in order to describe the breakthrough behavior of the system. These models were fitted to the experimental breakthrough curves, and the chi-square (*χ^2^*), Average Relative Error (*ARE*), and Marquardt’s Percent Standard Deviation (*MPSD*) error functions were used to assess the goodness of fit. In order to guarantee accurate interpretation of the column performance and to identify the best model to describe the system behavior, this study was carried out. The results of the modeling and error analysis are presented in Table 6.

As shown in Table 6, the Thomas, Yoon–Nelson, and BDST models showed much lower error values than the Adams–Bohart model, indicating a better fit to the experimental breakthrough curves. The poor performance of the Adams–Bohart model confirms that it is only suitable for the initial region. Therefore, the Thomas and Yoon–Nelson model was selected as the most appropriate model to describe the column behavior. The fact that a process is defined by a model indicates that the behavior of the column can now be reliably predicted mathematically. 

### 3.4. Characterization of the Bead Structure

#### 3.4.1. SEM and SEM–EDX Analysis

**Figure 7:**
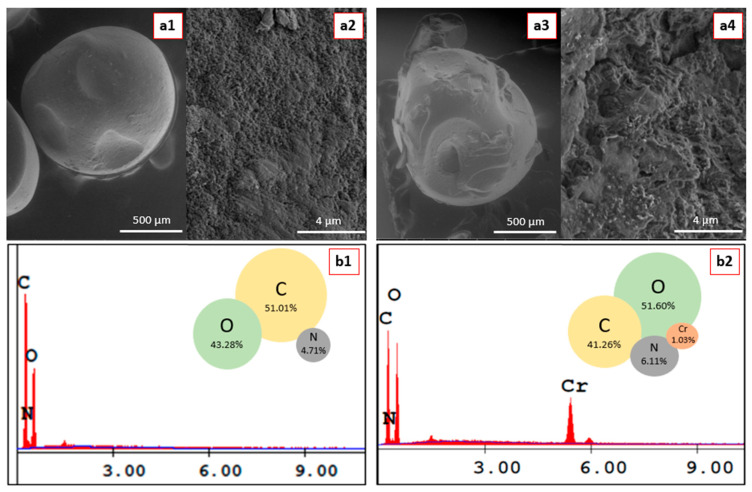
SEM images of (**a1**,**a2**) pristine and (**a3**,**a4**) Cr (VI)-loaded H-CS/PVA beads at different magnifications. SEM–EDX spectra of (**b1**) pristine and (**b2**) Cr (VI)-loaded H-CS/PVA beads.

SEM and SEM–EDX analyses were performed on unloaded and loaded samples to confirm the successful adsorption of Cr (VI) onto the adsorbent surface and to reveal the changes in surface morphology and elemental composition after adsorption. SEM and SEM–EDX analysis results for the pre-adsorption and post Cr (VI) adsorption of H-CS/PVA adsorbent are presented in Figure 7.

According to Figure 7(a1,a2), the surface of H-CS/PVA beads is characterized by numerous pores of varying sizes, configuring a rough and heterogeneous structure. Meanwhile, after Cr (VI) adsorption, bright spots were exposed on the surface, clearly indicating the presence of Cr (VI) ions (Figure 7(a3,a4)). The pores were clogged, and rather larger cavities were observed due to disrupted homogeneity.

SEM–EDX results (Figure 7(b1,b2)) showed that prior to adsorption, only C, N and O elements were present in the H-CS/PVA sample, whereas Cr appeared in the spectra after adsorption. Furthermore, increased elemental ratios of O and N, besides the decrease in the C ratio, indicated that Cr (VI) binds to the surface via oxygen and nitrogen-containing functional groups [55].

#### 3.4.2. XPS Analysis

**Figure 8:**
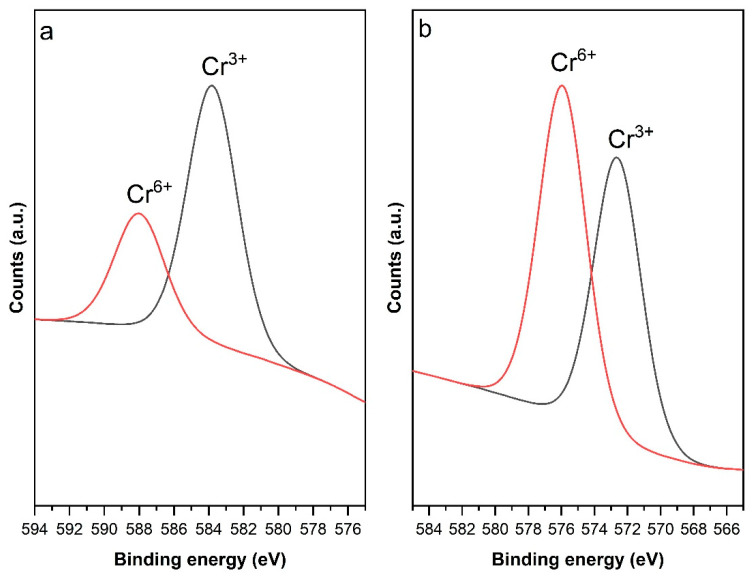
XPS analysis of H-CS/PVA adsorbent beads. (**a**) Cr2p_1/2_ and (**b**) Cr2p_3/2_ regions.

XPS analysis was performed on Cr-loaded H-CS/PVA adsorbent beads to further elucidate the adsorption mechanism of Cr ions on the adsorbent surface, the elemental composition of the CS-PVA beads, and chemical bonding states. The results are presented in Figure 8.

The spectral deconvolution of the Cr2p spectrum of the Cr-doped H-CS/PVA adsorbent beads, given in Figure 8, revealed that chromium is present as Cr^3+^ and Cr^6+^ states. Deconvolution was performed by fitting the Cr2p spectrum into its spin–orbit components, where the lower binding energy peaks correspond to Cr2p^3/2^ and the higher binding energy peaks to Cr2p^1/2^. The spin–orbit splitting between the Cr2p^3/2^ and Cr2p^1/2^ doublets was calculated to be in the range of 9.3 to 9.7 eV [56,57]. The coexistence of these chromium species suggests partial reduction of Cr^6+^ during the adsorption process, indicating that, besides adsorption, surface complexation, redox interaction and surface deposition mechanisms also prevail. Quantitative calculations based on the peak areas revealed that 53.74% of the total chromium on the surface was in the form of Cr^3+^ and 46.26% was Cr^6+^. The identification of Cr^3+^ as the dominant species in XPS demonstrates that the process proceeds via an “adsorption-coupled reduction” mechanism.

Generally, the mechanism of Cr (VI) adsorption upon various materials is described as a combination of electrostatic interactions, reduction and complexation mechanisms [39]. Under the studied pH conditions (i.e., 3.0), the prevailing HCrO4- ions and, partially, Cr_2_O_7_^2−^ are attracted by the protonated amine, carboxyl and hydroxyl functional groups [29,31]. These active sites can further reduce and form complexes with the adsorbed Cr ions by acting as electron donors [31]. According to this mechanism, toxic Cr^6+^ ions captured by functional groups on the surface of hydrothermally synthesized H-CS/PVA beads are reduced to a more stable and less toxic Cr^3+^ form [58].

#### 3.4.3. FTIR Analysis

**Figure 9:**
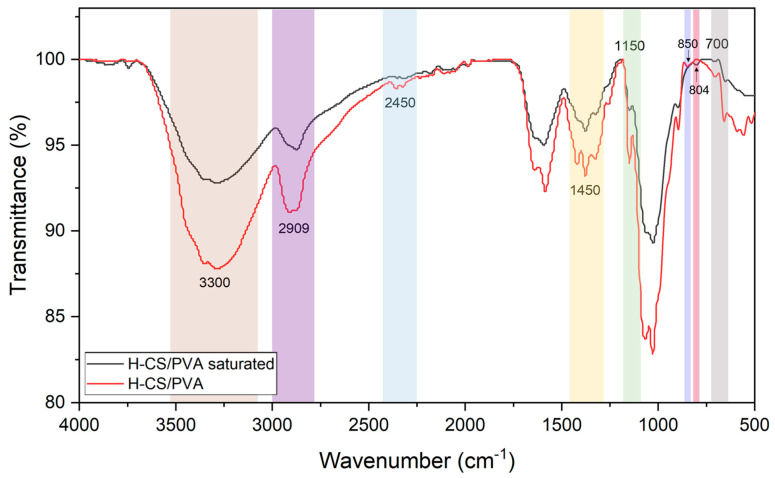
Comparative FTIR spectra of H−CS/PVA beads prior to and after saturation with Cr (VI).

The FTIR spectra of the H-CS/PVA beads obtained before and after the adsorption of H-CS/PVA are given in Figure 9.

In the FTIR spectra (Figure 9), the broad band around ~3300 cm^−1^ is attributed to –OH and –NH stretching vibrations, while the band at ~2900 cm^−1^ corresponds to C–H stretching [59]. The peaks observed in the range of ~1650–1450 cm^−1^ are associated with amide and N–H bending vibrations, and those between ~1150–1000 cm^−1^ are assigned to C–O and C–N stretching modes [60]. After adsorption of Cr ions, shifts and changes in intensity/shape were observed in the characteristic bands of the –OH and –NH groups. Moreover, characteristic peaks in the fingerprint region (700–500 cm^−1^) attributable to N-H and C-O out-of-plane bending were significantly suppressed after adsorption, indicating Cr-O interactions [61]. The shift of the band that appeared at ~850 cm^−1^ for unloaded beads to 804 cm^−1^ after adsorption was attributed to Cr (III)−OH vibrations and thus was accepted as an indication of Cr (VI) reduction [31,50]. These changes indicate that Cr ions interact directly with hydroxyl and amine groups, attaching to the H-CS/PVA surface, and that the adsorption reactions occurring on the surface are consistent with the spectroscopic changes observed in FTIR.

#### 3.4.4. BET Analysis

BET analyses were performed on both unloaded and loaded H-CS/PVA samples to determine the extent to which Cr (VI) filled the surface and pores by comparing changes in the specific surface area and pore structure of the samples before and after adsorption. The specific surface area and total pore volume of unloaded H-CS/PVA beads were 2.96 m^2^/g and 0.35 cm^3^/g, respectively. After Cr (VI) adsorption, both values decreased to nearly zero, indicating that the surface-active sites and pore structure were extensively occupied by Cr (VI) ions, which severely limited the accessibility of N_2_ molecules to the porous network. Similarly, Xavier et al. have acknowledged more than 80% reduction in the surface area and overall pore volume of heavy metal-loaded aluminosilicates [62]. Tang et al. have related the 50% decrease recorded in the BET surface area of mesoporous carbon after iron doping to the occupation and blocking of pores by the metal [63].

### 3.5. Regeneration Studies

**Figure 10:**
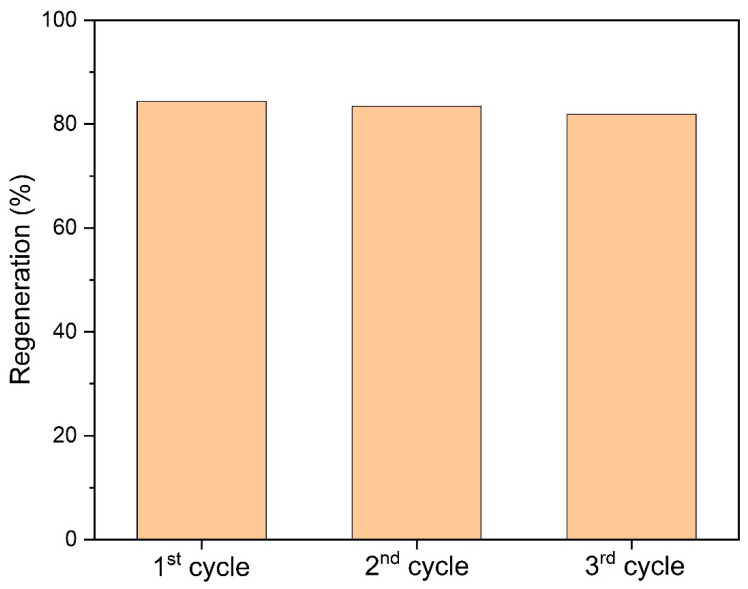
Adsorption–desorption study conducted under optimal column conditions.

Among the investigated desorption agents, i.e., EDTA, NaOH and HCl solutions, NaOH was identified as the eluent with the highest regenerative capacity. The results of the desorption experiment conducted under optimal column conditions are summarized in Figure 10.

The desorption process is influenced by a variety of parameters, with the most significant being the nature and composition of the desorption solution and also operational conditions such as temperature and pH [64]. The regeneration and reusability performance of the H-CS/PVA using 0.1 M NaOH showed only a slight decrease of approximately 2.5% over three consecutive cycles (Figure 10), outperforming the performance of a similar binary polymeric blend reported by Attia et al., which lost its adsorption capacity by 91% after three consecutive cycles [35]. In this context, the sustained desorption performance of the present material highlights its superior competitiveness amongst those documented in the literature.

### 3.6. Life Cycle Assessment

Life cycle assessment is recognized as a vital tool for evaluating a product’s environmental impact on a broader production scale, in addition to its cost and technological feasibility [65]. The primary objective of designing the column for H-CS/PVA beads is to test the material’s industrial suitability, and thus, a life cycle analysis is essential.

Under the optimum column conditions, i.e., a bed height of 6 cm and column diameter of 2.5 cm, approximately 10 g of adsorbent is required. For the production of this amount, PVA, CS, acetic acid, and NaOH were used as the main materials, with 5 g of each of the first three materials and 4 g of NaOH. The emission factors were 2.2, 1.64, 1.2, and 1.12 kg CO_2-_eq, respectively. In addition to 1 L of distilled water used for solution preparation, gelation, and bead washing processes, an electricity consumption of 16 kWh was estimated for autoclaving and general laboratory use. The corresponding emission factors are both 0.05 kg CO_2_-eq/kg [66,67,68]. Therefore, the total CO_2_-eq value of all consumables adds up to 0.879 kg CO_2_-eq for 20 g of H-CS/PVA synthesis. Overall, the desorption step was carried out using approximately 100 mL of 0.1 M NaOH solution; therefore, 0.4 g NaOH and 0.1 L of water were consumed, resulting in a total environmental burden of approximately 0.00545 kg CO_2_-eq. The total environmental impact associated with treating 20 L of wastewater using a system comprising three desorption cycles has been determined to be approximately 0.896 kg CO_2_-eq based on the specific limits and assumptions mentioned above. Within this framework, life cycle assessment (LCA) studies can be easily expanded and adapted by applying similar approaches to different scales.

In order to enable comparison with other treatment approaches from an LCA perspective, the results were normalized to a functional unit of kg CO_2_-eq per liter of treated water. Accordingly, 0.04395 kg CO_2_-eq/L was calculated with the highest carbon footprint originating from the electricity consumption (approximately 90% of total spending). This footprint is significantly lower than the footprint reported by Choudhary et al. (2024) [69], who remediated lead-, chromium- and copper-contaminated wastewater via an electrocoagulation method at a burden of 0.0628 kg CO_2_-eq/L due to high electricity utilization. Yet, it was possible to reduce the carbon impact to 0.0165 kg CO_2_-eq/L and 0.0112 kg CO_2_-eq/L with alternate energy sources, i.e., solar and wind, respectively [69]. The integration of such a strategy into the fabrication of H-CS/PVA beads would significantly reduce the footprint of the hydrothermal treatment and, consequently, the total environmental burden of fixed-bed column applications. It is important to note that comparisons between systems should be interpreted cautiously, as each system must be evaluated in detail regarding its specific boundaries and scale-up assumptions, which can significantly influence the results. Therefore, direct and comprehensive comparisons across different studies may be feasible depending on the availability of standardized data.

## 4. Conclusions

Hydrothermal carbonization was utilized to synthesize acid-resistant chitosan/polyvinyl alcohol adsorbent beads for the continuous adsorption of chromium. The data obtained in fixed-bed column studies showed that increasing the flow rate and inlet concentration shortened the breakthrough time, while increasing the bed height improved the removal efficiency. Under optimal conditions, the maximum removal efficiency reached 64.2%, and the maximum adsorption capacity was determined to be 15.53 mg/g. Though the capacity and removal efficiency of H-CS/PVA beads were moderate relative to the literature, potential improvements can be obtained by the incorporation of functional additives such as MWCNT, nanocellulose or graphene, which will form the scope of further research. Yet, the stable regeneration behavior over three repeated cycles using 0.1 M NaOH indicated the superior structural and functional stability of the H-CS/PVA beads. Moreover, LCA revealed that 20 L of Cr (VI)-contaminated wastewater could be treated at a burden of ~0.9 kg CO_2_-eq.

The experimental breakthrough curves were successfully analyzed using the Yoon–Nelson model due to the lowest error function values. The BDST model was found useful for column design purposes. Characterization of the beads with SEM–EDX, FTIR, BET and XPS methods proved the partial reduction of Cr (VI) to Cr (III) prior to adsorption. Conclusively, the results not only demonstrated the efficacy of hydrothermal treatment in the fabrication of acid-resistant CS/PVA adsorbent beads as a green alternative that eliminates chemical cross-linkers but also proved the promising potential of H-CS/PVA as a low-cost, sustainable biopolymeric packing material for Cr (VI) removal in fixed-bed adsorption systems, particularly.

## Figures and Tables

**Table 2:** Fixed-bed column parameters for different flow rates, initial concentrations and bed heights.

Varied Parameter	C_0_ (mg/L)	Q (mL/min)	h_bed_ (cm)	Mass (g)	t_b_ (min)	t_sat_ (min)	m_tot_ (mg)	q_tot_ (mg)	q_e_ (mg/g)	V_eff_ (mL)	Removal (%)
Flow rate	20	2.5	2	4.32	250	720	36	23.12	5.36	1800	64.2
	20	5	2	4.31	60	510	51	22.62	5.24	2550	44.36
	20	7.5	2	4.32	40	480	72	16.64	3.85	3600	23.12
Initial Cr (VI) concentration	20	2.5	2	4.32	250	720	36	23.12	5.36	1800	64.2
	40	2.5	2	4.32	25	540	54	27.94	6.47	1350	51.75
	60	2.5	2	4.33	20	390	58.5	35.14	8.11	975	60.06
Bed height	40	5	2	4.31	45	510	102	42.92	9.95	2550	49.98
	40	5	4	7.09	180	720	144	76.67	10.81	3600	53.24
	40	5	6	10.03	240	1380	276	155.86	15.53	6900	56.47

**Table 3:** Model constants for the uptake of Cr (VI) onto H-CS/PVA.

Model	Experimental Conditions			Kinetic Constants			Error	
Thomas	**C_0_** **(mg/L)**	**Q** **(mL/min)**	**h_bed_** **(cm)**	**k_Th_** **(mL/mg·min)**	**q_0_** **(mg/g)**	**t_b,cal_** **(min)**	**t_b,exp_** **(min)**	ε **(%)**
	20	2.5	2	0.59	5.45	263	250	5.20
	20	5	2	0.67	6.77	127	60	111.67
	20	7.5	2	0.80	8.11	96	40	140.00
	40	2.5	2	0.27	6.49	77	25	208.00
	60	2.5	2	0.25	6.93	54	20	170.00
	40	5	2	0.30	11.66	68	45	51.11
	40	5	4	0.28	13.07	267	180	48.33
	40	5	6	0.19	14.17	368	240	53.33
Adams– Bohart	**C_0_** **(mg/L)**	**Q** **(mL/min)**	**h_bed_** **(cm)**	**k_AB_** **(−10^−4^ L/mg·min)**	**N_0_** **(mg/L)**	**t_b,cal_** **(min)**	**t_b,exp_** **(min)**	ε **(%)**
	20	2.5	2	0.00032	9329.5	316	250	26.40
	20	5	2	0.00035	12,958.5	140	60	133.33
	20	7.5	2	0.00037	17,629.6	114	40	185.00
	40	2.5	2	0.00014	13,664.7	83	25	232.00
	60	2.5	2	0.00013	14,896.7	64	20	220.00
	40	5	2	0.00014	25,246.8	46	45	2.22
	40	5	4	0.00017	18,842.5	343	180	90.56
	40	5	6	0.000072	23,371.1	470	240	95.83
Yoon–Nelson	**C_0_** **(mg/L)**	**Q** **(mL/min)**	**h_bed_** **(cm)**	**k_YN_** **(L/min)**	τ **_cal_** **(min)**	τ **_exp_** **(min)**		ε **(%)**
	20	2.5	2	0.0106	470.3	480		2.02
	20	5	2	0.0131	291.8	310		5.87
	20	7.5	2	0.0161	233.5	238		1.89
	40	2.5	2	0.0108	281.1	300		6.30
	60	2.5	2	0.0151	199.7	200		0.15
	40	5	2	0.0120	251.2	270		6.96
	40	5	4	0.0113	463.2	470		1.45
	40	5	6	0.0063	711.1	701		1.44

**Table 4:** BDST model parameters for Cr (VI) adsorption onto H-CS/PVA beads.

C_0_ (mg/L)	Q (mL/min)	h_bed_ (cm)	k_BDST_ (mL/mg·min)	N_0_ (mg/L)
20	2.5	2	0.00053	6496
20	5	2	0.00067	401
20	7.5	2	0.00080	3226
40	2.5	2	0.00027	7766
60	2.5	2	0.00025	8275
40	5	2	0.00030	13,879
40	5	4	0.00028	12,797
40	5	6	0.00016	13,095

**Table 5:** BDST model parameters for the adsorption of Cr (VI) on H-CS/PVA.

C_t_/C_0_	a (min/cm)	b (min)	k_BDST_ (mL/mg·min)	N_0_ (mg/L)	Z_0_ (cm)	R^2^
0.1	97.5	166.7	0.013	24,075	1.7	0.995

**Table 6:** Comparison of error analysis of kinetic models applied to fixed-bed column models.

Parameters			Thomas			Adams–Bohart			Yoon–Nelson			BDST		
Q ^1^	$C_{0}$ ^2^	Z ^3^	$X^{2}$	ARE	MPSD	$X^{2}$	ARE	MPSD	$X^{2}$	ARE	MPSD	$X^{2}$	ARE	MPSD
2.5	20	2	3.1	43.2	0.60	23.1	68.4	0.79	3.1	43.2	0.60	3.1	43.2	0.68
5	20	2	2.8	23.9	0.39	20.0	45.6	0.58	2.8	23.9	0.39	2.8	23.9	0.51
7.5	20	2	4.2	30.5	0.46	31.9	58.1	0.69	4.2	30.5	0.46	4.2	30.5	0.58
2.5	40	2	15.8	33.8	0.48	47.3	54.0	0.63	15.8	33.8	0.48	15.8	35.2	0.62
2.5	60	2	30.4	38.9	0.52	70.3	54.9	0.66	30.4	38.9	0.52	30.4	38.9	0.66
5	40	2	14.8	31.8	0.47	48.8	53.8	0.64	14.8	31.8	0.47	14.8	31.8	0.59
5	40	4	4.2	45.8	0.58	36.3	68.1	0.77	4.2	45.8	0.58	4.2	45.9	0.71
5	40	6	3.9	20.1	0.30	58.5	53.8	0.64	3.9	20.1	0.30	3.9	24.8	0.48

^1^ In units of mL/min. ^2^ In units of mg/L. ^3^ In units of cm.

## Data Availability

The data that support the findings of this study are available from the corresponding author (Y.A.A.) upon reasonable request.
